# On the analysis of glycomics mass spectrometry data via the regularized area under the ROC curve

**DOI:** 10.1186/1471-2105-8-477

**Published:** 2007-12-12

**Authors:** Jingjing Ye, Hao Liu, Crystal Kirmiz, Carlito B Lebrilla, David M Rocke

**Affiliations:** 1 Department of Statistics, University of California, Davis, Davis, CA, 95616, USA; 2 Division of Biostatistics, Dan L. Duncan Cancer Center, Baylor College of Medicine, Houston, TX, 77030, USA; 3 Department of Chemistry, University of California, Davis, Davis, CA, 95616, USA; 4 Division of Biostatistics, University of California, Davis, Davis, CA, 95616, USA

## Abstract

**Background:**

Novel molecular and statistical methods are in rising demand for disease diagnosis and prognosis with the help of recent advanced biotechnology. High-resolution mass spectrometry (MS) is one of those biotechnologies that are highly promising to improve health outcome. Previous literatures have identified some proteomics biomarkers that can distinguish healthy patients from cancer patients using MS data. In this paper, an MS study is demonstrated which uses glycomics to identify ovarian cancer. Glycomics is the study of glycans and glycoproteins. The glycans on the proteins may deviate between a cancer cell and a normal cell and may be visible in the blood. High-resolution MS has been applied to measure relative abundances of potential glycan biomarkers in human serum. Multiple potential glycan biomarkers are measured in MS spectra. With the objection of maximizing the empirical area under the ROC curve (AUC), an analysis method was considered which combines potential glycan biomarkers for the diagnosis of cancer.

**Results:**

Maximizing the empirical AUC of glycomics MS data is a large-dimensional optimization problem. The technical difficulty is that the empirical AUC function is not continuous. Instead, it is in fact an empirical 0–1 loss function with a large number of linear predictors. An approach was investigated that regularizes the area under the ROC curve while replacing the 0–1 loss function with a smooth surrogate function. The constrained threshold gradient descent regularization algorithm was applied, where the regularization parameters were chosen by the cross-validation method, and the confidence intervals of the regression parameters were estimated by the bootstrap method. The method is called TGDR-AUC algorithm. The properties of the approach were studied through a numerical simulation study, which incorporates the positive values of mass spectrometry data with the correlations between measurements within person. The simulation proved asymptotic properties that estimated AUC approaches the true AUC. Finally, mass spectrometry data of serum glycan for ovarian cancer diagnosis was analyzed. The optimal combination based on TGDR-AUC algorithm yields plausible result and the detected biomarkers are confirmed based on biological evidence.

**Conclusion:**

The TGDR-AUC algorithm relaxes the normality and independence assumptions from previous literatures. In addition to its flexibility and easy interpretability, the algorithm yields good performance in combining potential biomarkers and is computationally feasible. Thus, the approach of TGDR-AUC is a plausible algorithm to classify disease status on the basis of multiple biomarkers.

## Background

With rapidly developing biotechnology, the use of high-throughput clinical laboratory data to detect disease conditions and predict patients' outcomes is becoming a reality for medical practice. These technologies include microarray, mass spectrometry applied to proteomics, and new imaging modalities, which have been engaged in research on detecting clinical disease, predicting patients' responses to different treatments and evaluating the prognosis of patients with disease [1].

Among those new biotechnologies, mass spectrometry (MS) is used increasingly for protein profiling in cancer research. The basic goal is to predict cancer on the basis of peptide/protein abundance from the MS data. Recent literatures on cancer classification using MS have identified some potential protein biomarkers in serum to distinguish cancer from normal samples (Baggerly *et al.*[2], Wagner *et al.*[3], Adam *et al.*[4]). However, sensitivity and reproducibility remains as a major concern in making the protein technology reliable [5].

As an alternative, glycomics is proposed as a new trend for biomarker detection at the end of 20th century. Glycomics is the study of glycans (oligosaccharides), and glycoproteins. Apweiler *et al.*[6] estimated that at least 50% of human proteins are glycosylated. Since glycans play crucial roles in cell communication and signalling events [7], they may be implicated in cancer. Compared to potential protein or peptide biomarkers, oligosaccharides are highly sensitive to biochemical environment and are more easily identified and quantified [8]. Therefore, in a study conducted in this lab, clinical glycomics is used to identify potential biomarkers for the early detection of ovarian cancer.

Ovarian cancer is one of the most deadly types of cancer among women [8]. Many investigators believe that early detection of ovarian cancer would improve the patients' survival. CA 125 is the only FDA approved biomarker for the early detection of ovarian cancer. However, it has unreasonably low sensitivity and specificity. For instance, only 50% women with Stage I ovarian cancer will have an elevated CA 125 and many benign conditions can cause elevated levels [8].

**Figure 1:**
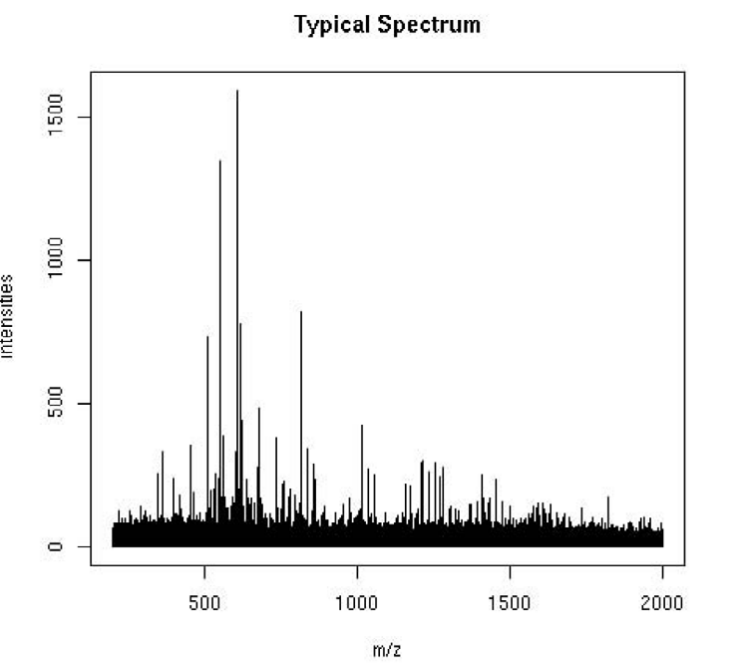
**Typical Glycomic Mass Spectrum**. A typical mass spectrometry glycomics spectra is plotted. The x-axis is m/z value and the y-axis is the corresponding intensity, which measures the relative abundance of glycans.

One technique of profiling oligosaccharides into serum was developed in this lab. The idea that glycoproteins which are sloughed off cells may be detected in patients' serum was utilized for this analysis. Serum samples were analyzed by the high-resolution MS to assess the variation of glycans in cancer patient sera compared to healthy patient sera. MS data are high-dimensional. Figure 1 shows a typical mass spectrum. In this experiment, a single spectrum contains 500,000 distinct mass-to-charge values (which measure the ratio of mass to the charge of glycans) and the corresponding relative intensities (which measure relative abundances of glycans) in the serum sample. It is desirable to use all informative glycans because multiple biomarkers may allow improved sensitivity and specificity of cancer detection [9].

A number of recent studies have implemented machine learning algorithms to classify high-dimensional MS cancer data. Artificial neural networks (ANNs) were utilized by Ball *et al.*[10], Lancashire *et al.*[11] and Mian *et al.*[12], to discriminate different tumor states. Fushiki et al. [13] explored the efficient learning algorithm AdaBoost to extract potential biomarkers for classifying MS cancer from control samples. Decision tree based ensemble methods were proposed by Geurts *et al.*[14] to identify biomarkers for inflammatory diseases. Other algorithms, such as support vector machine (SVM) used by Xiong *et al.*[15], random forest (RF) applied by Wu *et al.*[16] and linear discriminant analysis (LDA) employed by Miketova *et al.*[17] and Lilien *et al.*[18], were also studied. Comparisons among algorithms in a case study of ovarian cancer classification were evaluated by Datta and DePadilla [19] and Wu *et al.*[16]. All of the above studies aim to discover the potential MS biomarkers that can distinguish one group from another. However, for prognostic and diagnostic purposes, how to combine those MS biomarkers and whether or not the combination is optimal are not addressed in those studies. Thus, an objective of this research is to consider a statistical method that combines the high-dimensional MS measurements into a single score to classify cancer status jointly with suitable preprocessing of the data.

There are several studies on combining biomarkers. Su and Liu [20] studied the case where markers follow a multivariate normal distribution. They gave a closed form of optimal solution to the linear parameters. Normality is not suitable for mass spectrometry data because measurements of relative abundance are always positive. Pepe and Thompson [21] considered linearly combining two biomarkers by optimizing the area under the ROC curve. The method was developed only for low-dimensional situation. And it is not trivial to generalize the approach to high-dimensional case. Ma and Huang [22] applied Pepe and Thompson's idea of optimizing AUC to microarray experiment. They used multivariate normal distribution in the simulation study and assumed independence between biomarkers, which is not true for mass spectrometry data. In addition, they implemented the threshold gradient algorithm, first proposed by Friedman and Popescu [23], without correctly recognizing the regularization parameter. In this work, the question on how to combine the high-dimensional mass spectrometry predictors into a single score for the purpose of classification is addressed. The performance of a classifier by maximizing the area under the ROC curve for linearly combining the biomarkers is evaluated. The technical difficulty of this optimization problem is that the empirical AUC function is not differentiable. The objective function is in fact an empirical 0–1 loss function with a large number of linear predictors, and it is well known that such optimization problem is ill-imposed. An approach that regularizes the area under the ROC curve while replacing the 0–1 loss function with a sigmoid function was investigated. A constrained threshold gradient descent regularization algorithm, which is first introduced by Friedman and Popescu [23], to stabilize the estimates is applied. In Friedman and Popescu, they demonstrated their algorithm in a quadratic objective function. In this study, their objective function is replaced with the area under the ROC. A simulation is also conducted on mass spectrometry data under the case-control design that will generate joint distribution of diseased samples and normal samples to evaluate this algorithm.

The article is organized as the following. Simulation study is described in the Testing Section which describes how effective the proposed TGDR-AUC approach is. The Implementation Section is for real mass spectrometry ovarian cancer data analysis after a description of our preprocessing method. TGDR-AUC method is applied to low-dimensional and high-dimensional ovarian cancer data. In the Conclusion and Discussion Section, it is concluded that the TGDR-AUC algorithm is appropriate in the analysis of mass spectrometry glycomic data. A detailed description for TGDR-AUC algorithm is in Method Section. The definition and properties of the ROC curve are reviewed. The area under the ROC curve as the objective function for maximizing the performance of the classifier is proposed. Furthermore, several sigmoid functions that replace the 0–1 loss function are introduced and a simple comparison among the sigmoid functions is shown. Threshold gradient direct regularization algorithm is explained after selection of sigmoid function as well as the detailed algorithm for parameter estimations.

## Results

### Testing

Testing of the TGDR-AUC algorithm was demonstrated through a simulation study. Since the mass spectrometry measures the relative abundance of molecules, the measurement is always positive. Hence, a positive distribution is a reasonable choice for data simulation. In contrast to Ma and Huang [22], who simulated data under normal distribution and assumed independence among biomarkers, exponential distribution was chosen to generate the simulation data. Since a better classifier is desired, the data used for simulation were chosen so that the true AUC equals to 0.95. The simulation is generated as the following:

• Denote *X *as normal patient, and *m *is the number of normal patients. Denote *Y *as the disease patient, and *n *is the number of diseased patients. For simplicity, we choose *m *= *n*. The dimension of the biomarkers is denoted as *p*.

• Simulate *X*_*i *_as an exponential distribution with parameter *λ *= 1, *i *= 1, ..., *n*.

• Generate a Bernoulli trial *B*(1, 0.95) for *n *times.

• The data for the diseased patients are generated as *Y*_*i *_= *X*_*i *_+ 1 when Bernoulli trial is 1; *Y*_*i *_= *max*{0, *X*_*i *_- 1} when Bernoulli trail is 0.

The number of the replication was chosen to be 500. The data is generated as joint distribution of *X *and *Y*. The true probability is *P*(*X *<*Y*) = 0.95, no matter what the linear combination *β *is. The goal of the simulation study is to show that the maximizer of empirical AUC by TGDR regularization is in fact our targeted maximization problem (4) (see Additional file 1, 2 for examples of simulated data).

To study whether the ratio of *p *and *n *has any impact on the results, the simulation cases are considered for the different combination pair of *p *and *n *as *p/n *→ + ∞, *p/n *→ *c*, where *c *is a constant and *p/n *→ 0. The *p *and *n *pairs are (10,5),(10,10),(10,25),(10,50),(25,5), (25,10),(25,25),(25,50) and (50,5),(50,10),(50,25),(50,50). The data are partitioned randomly into a training set of size *n*_1 _and a testing size of *n*_2 _with *n*_1 _+ *n*_2 _= 2*n*. Dudoit [24] suggested that n1~232n≈1.3n
 MathType@MTEF@5@5@+=feaafiart1ev1aaatCvAUfKttLearuWrP9MDH5MBPbIqV92AaeXatLxBI9gBaebbnrfifHhDYfgasaacPC6xNi=xH8viVGI8Gi=hEeeu0xXdbba9frFj0xb9qqpG0dXdb9aspeI8k8fiI+fsY=rqGqVepae9pg0db9vqaiVgFr0xfr=xfr=xc9adbaqaaeGacaGaaiaabeqaaeqabiWaaaGcbaGaemOBa42aaSbaaSqaaiabigdaXaqabaGccqGG+bGFjuaGdaWcaaqaaiabikdaYaqaaiabiodaZaaakiabikdaYiabd6gaUjabgIKi7kabigdaXiabc6caUiabiodaZiabd6gaUbaa@3AA5@. The TGDR algorithm described in the Method Section was applied to the simulated data and summary statistics of estimated empirical AUC based on 500 simulated data sets are reported in Table 1.

**Table 1:** Simulation Study Result. Summary statistics of the simulation results of TGDR-AUC algorithm.

p	n	Bias	Mean of Empirical AUC	Median of AUC	Standard Error
10	5	0.0088	0.9588	1	0.0857
10	10	0.01674	0.96674	1	0.0535
10	25	0.0087	0.9587	0.96	0.0375
10	50	0.0009	0.9509	0.9576	0.0300
25	5	0.0166	0.9666	1	0.0779
25	10	0.0226	0.9726	1	0.047
25	25	0.016	0.966	0.968	0.0338
25	50	0.007	0.957	0.96	0.0273
50	5	0.0172	0.9672	1	0.0795
50	10	0.028	0.978	1	0.0473
50	25	0.0218	0.9718	0.9808	0.0311
50	50	0.0086	0.9586	0.96	0.0268

The simulation conducted is for a relatively small sample size and a small number of biomarkers. From Table 1, the regularization of TGDR tends to overestimate the AUC when *p *is less than *n*. As sample size increases, the estimated AUC by regularization approximates the true AUC. Furthermore, as sample size increases, the standard error stabilizes to be around 0.03.

Table 1 gives a guideline on how to use the TGDR-AUC algorithm for different situations. When a larger sample size *n *than biomarker *p *is observed, the algorithm is trustworthy, in the sense that the estimated AUC approximated the true AUC and the best ratio for *p/n *is around 1/5.0. However, when the number of biomarkers is much larger than the sample size, it remains unclear whether the TGDR-AUC algorithm tends to overestimate the AUC.

## Implementation

Two real MS data sets are analyzed, one low-dimensional and another high-dimensional real ovarian cancer data. The low-dimensional data is preprocessed, and high-dimensional data is the mass spectrometry raw data. So high-dimensional data will be preprocessed first before it is carried on any further analysis. The high-dimensional data is a subset of the low-dimensional data because of missing raw data files. The TGDR-AUC algorithm is applied to both data sets for cancer diagnosis. All of the programming were done in C and R (see Additional file 2, 3, 4 for algorithm codes).

### Low-dimensional Ovarian Cancer Data Analysis

**Figure 2:**
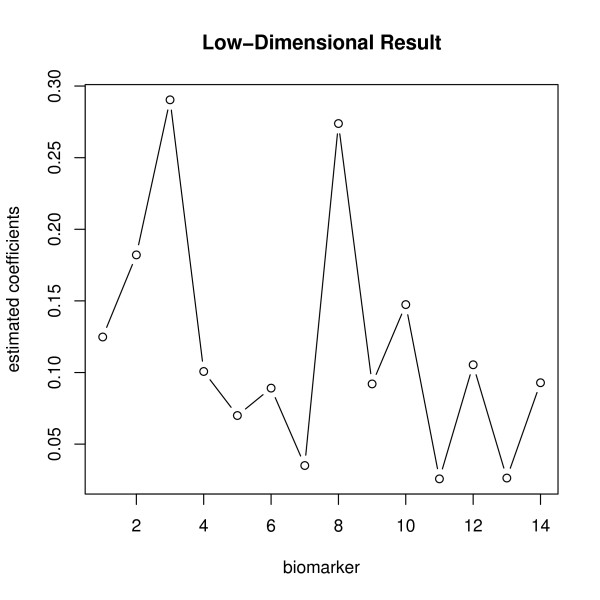
**Low-dimensional Data Result**. Plot of estimated coefficients by TGDR-AUC algorithm of potential biomarkers for Low-dimensional data.

**Figure 3:**
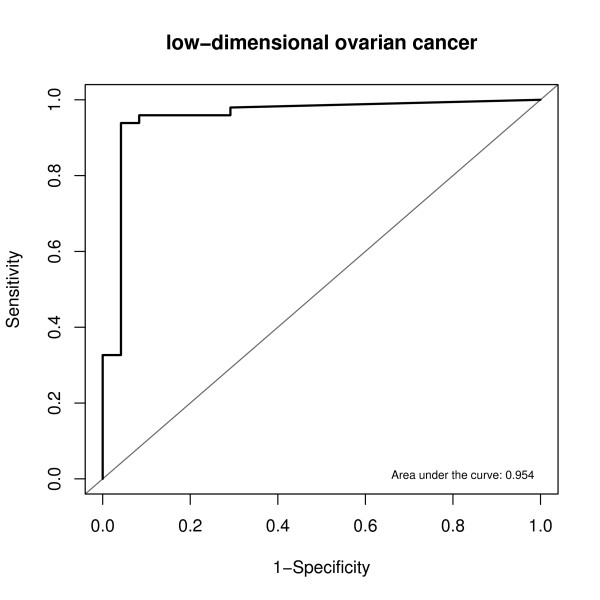
**Low-dimensional Data ROC**. Estimated ROC curve given the estimated optimal combination by TGDR-AUC algorithm of biomarkers for low-dimensional data.

The data contained 73 patients, among which 24 were healthy patients and 49 were ovarian cancer patients. Total 14 glycan biomarkers were pre-selected for the low-dimensional data set. The 3-fold cross-validation TGDR-AUC algorithm was applied to select regularization parameter *λ *and then select the optimal tuning parameter *τ*. Using the determined *λ *and *τ*, the estimated empirical AUC for the data was further obtained. The result is summarized in the first column in Table 2. The estimated empirical AUC was as high as 0.95, which indicates an excellent cancer diagnosis for linear combing the 14 biomarkers. The ratio of biomarker number 14 to sample size number 73 was less than 1/5.0, so the estimated empirical AUC was trustworthy as suggested by the simulation results. The 500 bootstrap data sets were performed for given both *λ *and *τ*. The bootstrap standard error (SE) was 0.00126. The 95% confident interval for AUC was (0.9513,0.9560). The estimated coefficients of the 14 biomarkers are plotted in Figure 2. From the figure, biomarkers number 3 and 8 had the highest coefficients, suggesting the highest influence on the cancer diagnosis. Biomarkers number 11 and 13 had smaller estimated values, indicating less importance than other biomarkers. The low-dimensional data ROC based on the 14 peaks are plotted in Figure 3.

**Table 2:** Ovarian Cancer Data (with the bootstrap standard error in parenthesis). Implementation results of TGDR-AUC algorithm to MS ovarian cancer data.

Estimators	Low-dimensional	High-dimensional
Empirical AUC	0.953656(0.00126)	0.994987(0.0002527)
*τ*	1	0.1
*λ*	0.081559	0.000436

### High-dimensional Ovarian Cancer Data Analysis

In this section, the analysis starts from the raw ovarian cancer data. The problem with the raw mass spectrometry data is that the data is high-dimensional, so extracting useful information is crucial. For this high-dimensional data set, there were 19 normal patients and 21 cancer patients. Each patient had three measurements, called 10%, 20% and 40% fractions, corresponding to different sample extraction methods carried out prior to the mass spectrometry experiments. For each spectrum, the raw data contained 500,000 data points. The mass spectrometer's manufacturer(Varian FTMS Systems, Lake Forest, CA) provided their software for peak-selection of individual spectra from the 500,000 data points. The problem with their peak-identification is that it is based on an individual spectrum, meaning that for any two spectra, peaks are selected at different mass-to-charge values. Therefore, the peaks are not consistent between samples and not trustworthy for cancer diagnosis. Before any data analysis is performed, the preprocessing of the data is critical.

### Preprocessing High-Dimensional Data

First, the peaks selected by the instrument's software for the spectra were used. The selected-peaks were grouped into a matrix, with each column corresponding to one spectrum and each row corresponding to one distinct mass-to-charge (m/z) value. If the spectrum did not have the peak at the m/z value, a zero was replaced to indicate missing value in the matrix. However, this resulted in many zeros in the matrix. The corresponding raw data was chosen to be substituted into the zero intensity. In this way, much more similar information could be included as the raw data.

However, the scale of raw data was not the same as the corresponding selected-peaks data file. The ratio factor between the raw and its corresponding selected-peaks file needed to be estimated. The estimation of the ratio factor was done as the following: find the nearest point in the raw data to its corresponding selected-peaks data, defined as the absolute distance between the corresponding m/z values. Two cases may happen: if the nearest m/z value in the raw data is unique, the ratio is calculated by the raw data intensity to the selected-peaks data intensity at the nearest value; if the nearest value is not unique, the ratio is calculated by the averaged intensities of raw data at those nearest values to the selected-peaks data intensity. For each file, the ratio was then averaged to give the unique factor estimation.

Using the estimated ratio for each file, the intensities from raw data could then be calculated to fill in the data matrix. There, again, may be two scenarios: if the closest m/z in raw data is unique to the selected-peaks data, then substitute in the corresponding raw data intensity with the adjustment by its ratio factor; if the closest m/z in raw data is not unique, then substitute in the maximum intensities of those closest m/z raw data to the corresponding column of the data matrix, with adjusting by its ratio factor. This was chosen as the maximum intensity because we wanted to include the strongest signal to be substituted in the data matrix.

After imputing the data as above, the data matrix was formed with each spectrum as one column of the matrix and intensities at all same m/z values. Before any statistical analysis could be completed, each column of the data matrix was further normalized by dividing total ion current of the corresponding raw data intensities to make sure the comparison of the spectrum would be made on the same level. An arbitrary factor 100000 times the intensities in the data matrix was to amplify the normalized intensities to a reasonable magnitude. Because the data variation was dependent on the mean, log transform was carried out on the data matrix. An arbitrary 0.00000001 was added in the intensities to ensure valid log transformation. The 10%, 20% and 40% fractions were combined by adding intensities up at each m/z value to group the data into one patient as one column in the data matrix.

After appropriate preprocessing of normalization and log transformation, the intensities for MS data are assumed to approximately meet the t-test requirements. We then performed a t-test to each m/z value on factor whether the patient had cancer or not. The p-values of the tests were recorded. The false discovery rate (FDR) by Benjamini and Hochberg [25] was applied to the t-test p-values to adjust to multi-test problem. Only adjusted p-values less than 0.05 were selected out to be potential biomarkers. As a result, 1228 biomarkers were selected for the high-dimensional data case.

### High-dimensional Data analysis result

A 3-fold cross-validation of the TGDR-AUC algorithm was applied to the data. The result is listed as the second column in Table 2. The estimated empirical AUC was almost perfect, close to 1. The bootstrap method was applied to estimate the empirical AUC confident interval for 1228 biomarkers. 500 bootstrap data sets were generated. The bagged empirical AUC for the given optimal pair of *λ *and *τ *was calculated from the bootstrap sample. The estimated standard error was 0.0002527. The confident interval for bootstrap was (0.9945, 0.9955). The high-dimensional data had a small sample size compared to a large number of biomarkers, which suggests that the estimated empirical AUC may be overestimated by the simulation.

**Figure 4:**
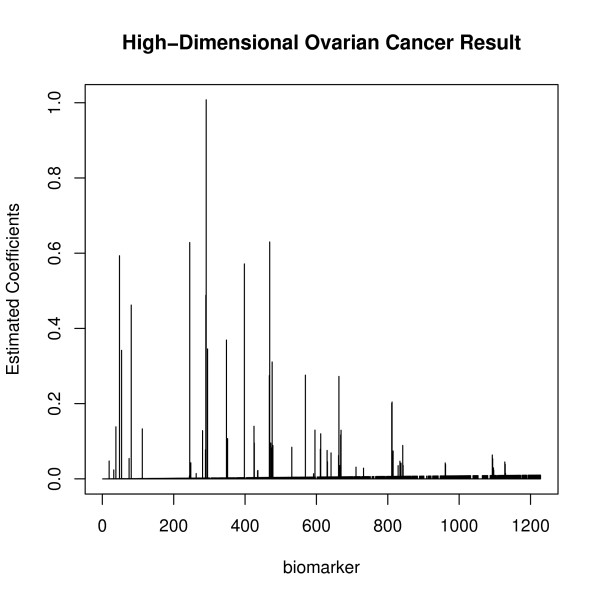
**High-dimensional Data Result**. Plot of estimated coefficients by TGDR-AUC algorithm of potential biomarkers for high-dimensional data.

**Figure 5:**
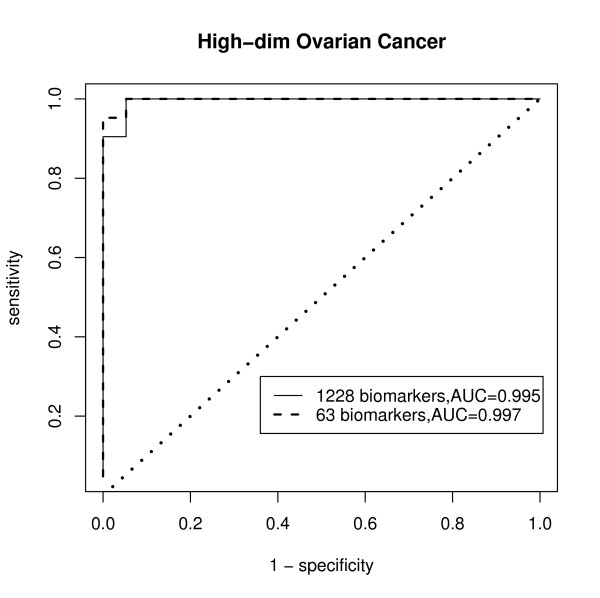
**High-dimensional Data ROC**. Estimated ROC curve given the estimated optimal combination by TGDR-AUC algorithm of biomarkers for high-dimensional data.

The estimated coefficients were also of interest, and the estimated coefficients are plotted in Figure 4. There were 139 biomarkers (more than 10% of total biomarkers) that had zero coefficients. Only 63 biomarkers had larger than 0.01 estimated coefficients. The TGDR-AUC algorithm provided a simultaneous dimension reduction technique so that if the estimated coefficient was zero, the corresponding biomarker did not have contribution to the AUC optimization. In this case, the dimension was reduced to 5% of the original dimension of 1228. McIntosh and Pepe [26] mentioned in their work that the AUC increases with the number of combined biomarkers. However, this may not be true. To see this, only the 63 biomarkers that have larger estimated coefficients were chosen and all the rest biomarkers coefficients were set to be zero. The ROC using all estimated 1228 biomarkers was compared to the ROC using only 63 biomarkers in Figure 5. The ROC with 63 biomarkers had a higher AUC value compared to AUC using all 1228 biomarkers. Although there was earlier doubt about the empirical AUC being optimistic, the resulting empirical AUC with smaller biomarker numbers indicated that this was a valid approach. Therefore, TGDR-AUC algorithm is a good classifier that provides the sufficiently unbiased AUC. Selected biomarker lists were further compared to those of peaks selected by their biochemical properties. Those 63 biomarkers were used because of their larger estimated coefficients, which indicated their potential as cancer biomarkers. Table 3 lists this result. The oligosaccharide composition was from Hyun Joo An, *et al.*[8]. The observed masses were also from that work for comparison. The biomarkers selected in that study matched well to those in this analysis. All of these biomarkers had high positive coefficients, which again suggest their potential contribution to cancer identification. More peaks of biomarkers are detected by more objective optimization method of regularized AUC.

**Table 3:** Comparisons of selected biomarkers between Hyun Joo An, et al. [8] and the TGDR-AUC algorithm. The biomarkers in Hyun Joo An, et al. [8] were selected based on their biochemical properties.

Observed Mass	Oligosaccharide Composition	Estimated linear coefficient
347.10	2Hex	0.138
388.14	1HexNAc:1Hex	0.462
509.17	3Hex	0.628
550.21	1HexNAc:2Hex	0.077
712.28	3Hex:1HexNAc	0.6299
772.31	2Hex:1HexNAc:1Hex	0.084
874.36	4Hex:1HexNAc	0.069
915.38	3Hex:2HexNAc	0.2724
975.43	2Hex:2HexNAc:1Hex	0.031
1077.47	4Hex:2HexNAc	0.204
1137.51	3Hex:2HexNAc:1Hex	0.0888
1280.62	4Hex:3HexNAc	0.0424
1442.72	5Hex:3HexNAc	0.0635
1502.74	4Hex:3HexNAc:1Hex	0.0449

**Figure 6:**
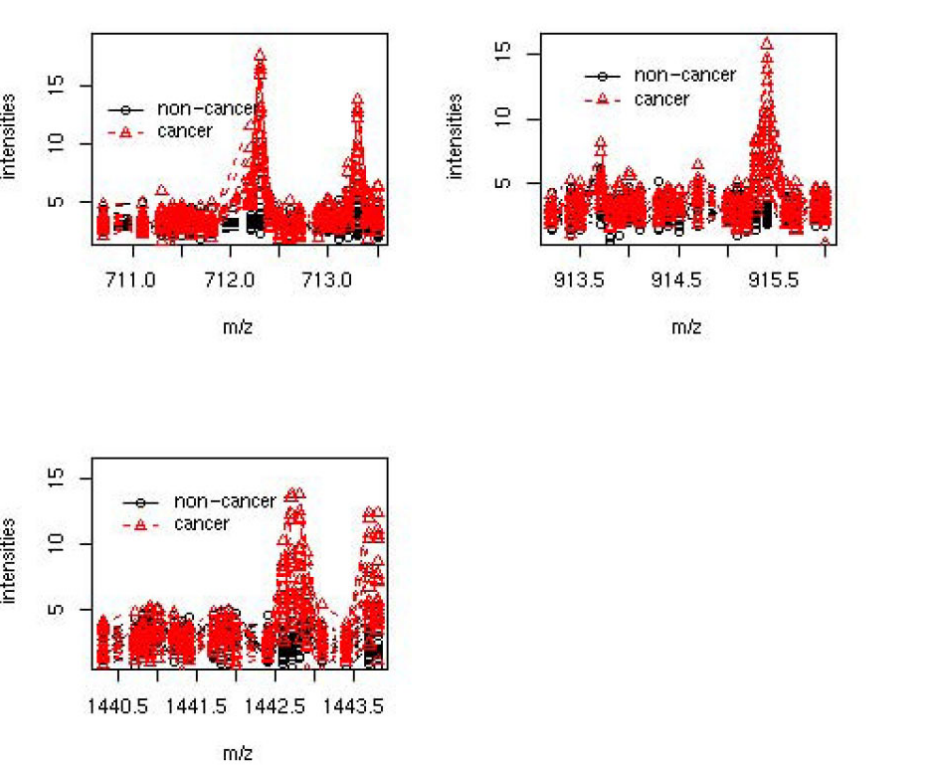
**Three m/z value area to distinguish cancer from non-cancer**. Plot of m/z values areas which can discriminate ovarian cancer (red) from non-cancer (black). The upper left plot is for m/z value 712.28; the upper right plot is for m/z 915.38 and lower plot is for m/z 1442.72. The m/z values are visually larger for cancer patients. For quantification purpose, simple descriptive statistics for the three areas are reported as the following: for m/z value 712.28, mean(standard deviation) of non-cancer samples are 4.506(3.181) and cancer samples are 10.633(3.615). The FDR-adjusted p-value for the comparison is 7.827e-07; for m/z value 915.38, mean(standard deviation) of non-cancer samples are 3.667(2.614) comparing to cancer samples 8.443(3.307). This results in FDR-adjusted p-value of 7.642e-06. The higher m/z 1442.72 has 2.958(2.435) for non-cancer samples of mean(standard deviation), while cancer samples are 7.275(3.1). The FDR-adjusted p-value for high mass area is 1.841e-05.

Three m/z values with large positive coefficients are plotted in Figure 6. The *m/z *values 712.28, 915.43 were selected because of their relative large estimated coefficients and *m/z *value 1442.72 was illustrated for a higher mass range. The three plots correspond to the three areas. Black is for healthy patients and red for cancer patients. From the Figure 6, all of the areas visually showed larger intensities for cancer patients than healthy patients. Larger coefficients had visually larger differences between the groups. The estimations were verified to make biological sense.

## Conclusion and Discussion

The key contribution of this work is that the optimal rules, for purpose of classifying disease status on the basis of multiple biomarkers, are based on the maximization of the area under the ROC subjected to constrained threshold gradient direct algorithm. The approach presented here relaxes the normality assumption and the approach of Pepe and Thompson [21] is generalized. The analysis is applied to the high-dimensional mass spectrometry glycomics data. In contrast to Ma and Huang [22], the simulation data is generated based on the joint distribution of disease samples and control samples with non-normality assumption, which is more appropriate for mass spectrometry data. This simulation is able to assess the difference between the maximization of the empirical AUC and the target AUC. The simulation proves the asymptotic properties that estimated TGDR-AUC approaches the true AUC when the sample size is increasing compared to the dimensionality of *p *biomarkers. When applied to the real ovarian cancer data, the algorithm also provides the build-in dimension reduction technique. For the high-dimensional ovarian cancer data, we can detect the 63 most important biomarkers among the total of 1228 biomarkers with simultaneously estimating the linear combination coefficients. The selected 63 biomarkers match very well with the 14 peaks pre-selected based on biological evidence. The algorithm is a non-parametric approach, very flexible and easy to interpret. The algorithm is aimed to have optimal classification. The resulting AUC of the linear combination is plausible and should be optimal among all other possible combinations. The computation of TGDR-AUC is computational feasible of high-dimensional data. This high-dimensional analysis evaluates more than 1000 biomarkers in the algorithm and essentially could consider more.

Although the result of simulation cannot guarantee the estimated AUC comes close enough to the true AUC in large *p *biomarker number for small sample size situation, the algorithm still provides enough information in recognizing potential biomarkers. Since the performance of small dimension of biomarkers in the large sample size scenario gives excellent overall result, iteratively combining the biomarkers in high-dimensional and reducing the dimension of biomarker to some reasonable size is considered. With the reduced dimension, the biomarkers are combined again using the TGDR-AUC algorithm. The algorithm would continue until the ratio of number of biomarker and the sample size are in the comfortable zone suggested by our simulation. The TGDR-AUC algorithm proves to be a promising algorithm and might be recommended in combining mass spectrometry biomarker analysis for cancer diagnosis.

## Methods

### ROC Curve

A case-control study is considered where the main outcome is binary denoted as *D*, where *D *= 1 as the case and *D *= 0 as the control. Denote the relative abundance of the *p *glycomics *R*_*p *× 1 _= (*R*_1_, ..., *R*_*p*_)^*T*^. We consider the linear combination score of the form

*L*_*β*_(*R*) = *β'R *= *β*_1_*R*_1 _+ *β*_2_*R*_2 _+ ... + *β*_*p*_*R*_*p*_

where β1×pT=(β1,...,βp)
 MathType@MTEF@5@5@+=feaafiart1ev1aaatCvAUfKttLearuWrP9MDH5MBPbIqV92AaeXatLxBI9gBaebbnrfifHhDYfgasaacPC6xNi=xH8viVGI8Gi=hEeeu0xXdbba9frFj0xb9qqpG0dXdb9aspeI8k8fiI+fsY=rqGqVepae9pg0db9vqaiVgFr0xfr=xfr=xc9adbaqaaeGacaGaaiaabeqaaeqabiWaaaGcbaacciGae8NSdi2aa0baaSqaaiabigdaXiabgEna0kabdchaWbqaaiabdsfaubaakiabg2da9iabcIcaOiab=j7aInaaBaaaleaacqaIXaqmaeqaaOGaeiilaWIaeiOla4IaeiOla4IaeiOla4IaeiilaWIae8NSdi2aaSbaaSqaaiabdchaWbqabaGccqGGPaqkaaa@4074@ is an unknown *p*-vector parameter and *R *serves as the classification predictors. The classification rule is constructed by *β'R*. To be more specific, we classify *D *= 1 if *β'R *≥ *c *and *D *= 0 otherwise, for a cutoff value *c*. By varying the cutoff value *c*, we obtain the Receiver Operating Characteristic(ROC) curve.

ROC is a graphical plot of the sensitivity and 1-specificity, also known as true positive rate (TPR) and false positive rate (FPR), respectively. The TPR and FPR are defined by

*TPR*(*c*) = *Pr*(*β'R *≥ *c*|*D *= 1),

*FPR*(*c*) = *Pr*(*β'R *≥ *c*|*D *= 0)

for any cutoff value *c*. By varying the discrimination value of *c*, the TPR and FPR are plotted to generate the ROC curve, which is a two-dimensional plot of FPR(*c*) vs TPR(*c*) with -∞ ≤ *c *≤ +∞. There is a balance between TPR and FPR. A completely random predictor would give a straight line at an angle of 45 degrees from the horizontal, from bottom left to top right, because as the threshold is raised, there would be equal numbers of true and false positives. ROC above the no-discrimination line would be preferred with better classification as the line closer to the upper left-corner point (0,1).

The overall performance of the classifier can be evaluated by the area under the ROC curve (AUC). Denote *n *as the number of diseased samples, *m *as the number of normal samples and *p *as the dimension of biomarkers. Denote *X*_*i *_= (*X*_*i*1_, ..., *X*_*ip*_) as the *i*-th normal subject, and *Y*_*j *_= (*Y*_*j*1_, ..., *Y*_*jp*_) as the *j*-th diseased subject, *i *= 1, ..., *m*, *j *= 1, ..., *n*. For a given parameter *β*, the corresponding ROC curve is generated by linearly combining the *p *biomarkers for classifier *β'Y *or *β'X*. It has been shown by Bamber [27] that the theoretical area under the ROC curve is a probability *Pr*(*β'Y *- *β'X *≥ 0). To achieve the optimal performance, we need to maximize

max⁡βPr(β′Y−β′X≥0).
 MathType@MTEF@5@5@+=feaafiart1ev1aaatCvAUfKttLearuWrP9MDH5MBPbIqV92AaeXatLxBI9gBaebbnrfifHhDYfgasaacPC6xNi=xI8qiVKYPFjYdHaVhbbf9v8qqaqFr0xc9vqFj0dXdbba91qpepeI8k8fiI+fsY=rqGqVepae9pg0db9vqaiVgFr0xfr=xfr=xc9adbaqaaeGacaGaaiaabeqaaeqabiWaaaGcbaWaaCbeaeaacyGGTbqBcqGGHbqycqGG4baEaSqaaGGaciab=j7aIbqabaacbiGccqGFqbaucqGFYbGCcqGGOaakcuWFYoGygaqbaiabdMfazjabgkHiTiqb=j7aIzaafaGaemiwaGLaeyyzImRaeGimaaJaeiykaKIaeiOla4caaa@40C4@

Statistically, the empirical AUC is given by

AUC(β)=1mn∑i=1m∑j=1nΨ(β;Xi,Yj),
 MathType@MTEF@5@5@+=feaafiart1ev1aaatCvAUfKttLearuWrP9MDH5MBPbIqV92AaeXatLxBI9gBaebbnrfifHhDYfgasaacPC6xNi=xI8qiVKYPFjYdHaVhbbf9v8qqaqFr0xc9vqFj0dXdbba91qpepeI8k8fiI+fsY=rqGqVepae9pg0db9vqaiVgFr0xfr=xfr=xc9adbaqaaeGacaGaaiaabeqaaeqabiWaaaGcbaGaemyqaeKaemyvauLaem4qamKaeiikaGccciGae8NSdiMaeiykaKIaeyypa0tcfa4aaSaaaeaacqaIXaqmaeaacqWGTbqBcqWGUbGBaaGcdaaeWbqaamaaqahabaGaeuiQdKLaeiikaGIae8NSdiMaei4oaSJaemiwaG1aaSbaaSqaaiabdMgaPbqabaGccqGGSaalcqWGzbqwdaWgaaWcbaGaemOAaOgabeaakiabcMcaPaWcbaGaemOAaOMaeyypa0JaeGymaedabaGaemOBa4ganiabggHiLdaaleaacqWGPbqAcqGH9aqpcqaIXaqmaeaacqWGTbqBa0GaeyyeIuoakiabcYcaSaaa@535B@

where the function Ψ(*β*; *X*_*i*_, *Y*_*j*_) in (5) is defined as

Ψ(β;Xi,Yj)={1,ifβ′Yj−β′Xi>0,12,ifβ′Yj−β′Xi=00,ifβ′Yj−β′Xi<0.
 MathType@MTEF@5@5@+=feaafiart1ev1aaatCvAUfKttLearuWrP9MDH5MBPbIqV92AaeXatLxBI9gBaebbnrfifHhDYfgasaacPC6xNi=xI8qiVKYPFjYdHaVhbbf9v8qqaqFr0xc9vqFj0dXdbba91qpepeI8k8fiI+fsY=rqGqVepae9pg0db9vqaiVgFr0xfr=xfr=xc9adbaqaaeGacaGaaiaabeqaaeqabiWaaaGcbaGaeuiQdKLaeiikaGccciGae8NSdiMaei4oaSJaemiwaG1aaSbaaSqaaiabdMgaPbqabaGccqGGSaalcqWGzbqwdaWgaaWcbaGaemOAaOgabeaakiabcMcaPiabg2da9maaceqabaqbaeaabmabaaaabaGaeGymaedabaGaeiilaWcabaGaeeyAaKMaeeOzaygabaGaf8NSdiMbauaacqWGzbqwdaWgaaWcbaGaemOAaOgabeaakiabgkHiTiqb=j7aIzaafaGaemiwaG1aaSbaaSqaaiabdMgaPbqabaGccqGH+aGpcqaIWaamcqGGSaalaKqbagaadaWcaaqaaiabigdaXaqaaiabikdaYaaaaOqaaiabcYcaSaqaaiabbMgaPjabbAgaMbqaaiqb=j7aIzaafaGaemywaK1aaSbaaSqaaiabdQgaQbqabaGccqGHsislcuWFYoGygaqbaiabdIfaynaaBaaaleaacqWGPbqAaeqaaOGaeyypa0JaeGimaadabaGaeGimaadabaGaeiilaWcabaGaeeyAaKMaeeOzaygabaGaf8NSdiMbauaacqWGzbqwdaWgaaWcbaGaemOAaOgabeaakiabgkHiTiqb=j7aIzaafaGaemiwaG1aaSbaaSqaaiabdMgaPbqabaGccqGH8aapcqaIWaamcqGGUaGlaaaacaGL7baaaaa@6EE6@

The empirical AUC is the same as the form of Mann-Whitney test statistics. The optimal estimator β^
 MathType@MTEF@5@5@+=feaafiart1ev1aaatCvAUfKttLearuWrP9MDH5MBPbIqV92AaeXatLxBI9gBaebbnrfifHhDYfgasaacPC6xNi=xH8viVGI8Gi=hEeeu0xXdbba9frFj0xb9qqpG0dXdb9aspeI8k8fiI+fsY=rqGqVepae9pg0db9vqaiVgFr0xfr=xfr=xc9adbaqaaeGacaGaaiaabeqaaeqabiWaaaGcbaacciGaf8NSdiMbaKaaaaa@2D8B@ is then defined as the maximizer of *AUC*(*β*).

### The Sigmoid Function

The main problem of the maximization (5) is that the objective function is not continuous, and thus not differentiable. The maximization is difficult to achieve and the maximizer is not unique. To overcome the difficulty, a smooth sigmoid function was chosen to approximate the objective function. The sigmoid function is a monotonically increasing function with a parameter *r *> 0 and lim_*x *→ -∞ _*S*_*r*_(*x*) = 0, and lim_*x *→ +∞ _*S*_*r*_(*x*) = 1. There are many choices of sigmoid functions, including:

S1,r(x)=tanh⁡(rx)+12,S2,r(x)=arctan⁡(rx)π+12,S3,r(x)=Pr[X≤rx]=∫−∞rx12πexp(−u22)du.
 MathType@MTEF@5@5@+=feaafiart1ev1aaatCvAUfKttLearuWrP9MDH5MBPbIqV92AaeXatLxBI9gBaebbnrfifHhDYfgasaacPC6xNi=xI8qiVKYPFjYdHaVhbbf9v8qqaqFr0xc9vqFj0dXdbba91qpepeI8k8fiI+fsY=rqGqVepae9pg0db9vqaiVgFr0xfr=xfr=xc9adbaqaaeGacaGaaiaabeqaaeqabiWaaaGcbaqbaeqabmqaaaqaaiabdofatnaaBaaaleaacqaIXaqmcqGGSaalcqWGYbGCaeqaaOGaeiikaGIaemiEaGNaeiykaKIaeyypa0tcfa4aaSaaaeaacyGG0baDcqGGHbqycqGGUbGBcqGGObaAcqGGOaakcqWGYbGCcqWG4baEcqGGPaqkcqGHRaWkcqaIXaqmaeaacqaIYaGmaaGccqGGSaalaeaacqWGtbWudaWgaaWcbaGaeGOmaiJaeiilaWIaemOCaihabeaakiabcIcaOiabdIha4jabcMcaPiabg2da9KqbaoaalaaabaGagiyyaeMaeiOCaiNaei4yamMaeiiDaqNaeiyyaeMaeiOBa4MaeiikaGIaemOCaiNaemiEaGNaeiykaKcabaacciGae8hWdahaaOGaey4kaSscfa4aaSaaaeaacqaIXaqmaeaacqaIYaGmaaGccqGGSaalaeaacqWGtbWudaWgaaWcbaGaeG4mamJaeiilaWIaemOCaihabeaakiabcIcaOiabdIha4jabcMcaPiabg2da9Gqaciab+bfaqjab+jhaYjabcUfaBjabdIfayjabgsMiJkabdkhaYjabdIha4jabc2faDjabg2da9maapedabaqcfa4aaSaaaeaacqaIXaqmaeaadaGcaaqaaiabikdaYiab=b8aWbqabaaaaOGaemyzauMaemiEaGNaemiCaahaleaacqGHsislcqGHEisPaeaacqWGYbGCcqWG4baEa0Gaey4kIipakiabcIcaOiabgkHiTKqbaoaalaaabaGaemyDau3aaWbaaeqabaGaeGOmaidaaaqaaiabikdaYaaakiabcMcaPiabdsgaKjabdwha1jabc6caUaaaaaa@8ED6@

**Figure 7:**
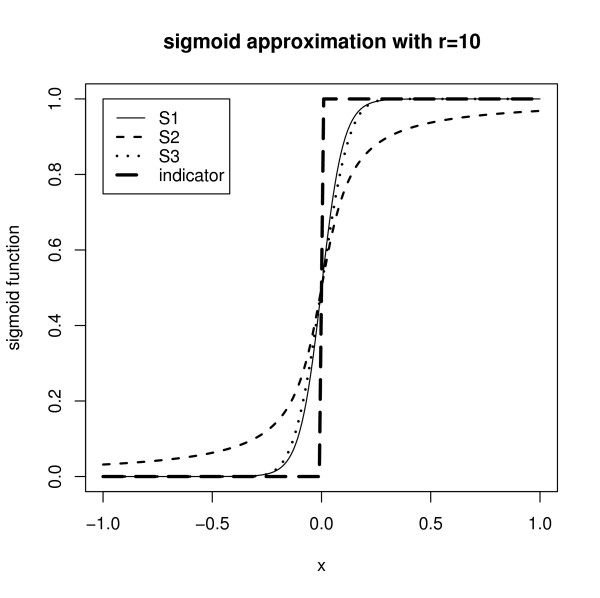
**Sigmoid Function Approximation with r = 10**. Several candidate sigmoid functions are plotted to approximate the indicator function.

The larger *r *is, the better the approximation will be. For a given value *r *= 10, Figure 7 shows the approximation result. The first function *S*_1, *r*_(*x*) approximates the best. The first function *S*_1, *r*_(*x*) can be simply written as

S1,r(x)=11+exp⁡(−2rx).
 MathType@MTEF@5@5@+=feaafiart1ev1aaatCvAUfKttLearuWrP9MDH5MBPbIqV92AaeXatLxBI9gBaebbnrfifHhDYfgasaacPC6xNi=xI8qiVKYPFjYdHaVhbbf9v8qqaqFr0xc9vqFj0dXdbba91qpepeI8k8fiI+fsY=rqGqVepae9pg0db9vqaiVgFr0xfr=xfr=xc9adbaqaaeGacaGaaiaabeqaaeqabiWaaaGcbaGaem4uam1aaSbaaSqaaiabigdaXiabcYcaSiabdkhaYbqabaGccqGGOaakcqWG4baEcqGGPaqkcqGH9aqpjuaGdaWcaaqaaiabigdaXaqaaiabigdaXiabgUcaRiGbcwgaLjabcIha4jabcchaWjabcIcaOiabgkHiTiabikdaYiabdkhaYjabdIha4jabcMcaPaaakiabc6caUaaa@43ED@

Therefore, for the choice of sigmoid function, the first function was used for further analysis. We now refer to the estimator β^
 MathType@MTEF@5@5@+=feaafiart1ev1aaatCvAUfKttLearuWrP9MDH5MBPbIqV92AaeXatLxBI9gBaebbnrfifHhDYfgasaacPC6xNi=xH8viVGI8Gi=hEeeu0xXdbba9frFj0xb9qqpG0dXdb9aspeI8k8fiI+fsY=rqGqVepae9pg0db9vqaiVgFr0xfr=xfr=xc9adbaqaaeGacaGaaiaabeqaaeqabiWaaaGcbaacciGaf8NSdiMbaKaaaaa@2D8B@ as the maximizer of

β^≡argmaxβ{1mn∑i=1m∑j=1nSr(β′(Yj−Xi))},
 MathType@MTEF@5@5@+=feaafiart1ev1aaatCvAUfKttLearuWrP9MDH5MBPbIqV92AaeXatLxBI9gBaebbnrfifHhDYfgasaacPC6xNi=xI8qiVKYPFjYdHaVhbbf9v8qqaqFr0xc9vqFj0dXdbba91qpepeI8k8fiI+fsY=rqGqVepae9pg0db9vqaiVgFr0xfr=xfr=xc9adbaqaaeGacaGaaiaabeqaaeqabiWaaaGcbaacciGaf8NSdiMbaKaacqGHHjIUcqWGHbqycqWGYbGCcqWGNbWzcqWGTbqBcqWGHbqycqWG4baEdaWgaaWcbaGae8NSdigabeaakmaacmqabaqcfa4aaSaaaeaacqaIXaqmaeaacqWGTbqBcqWGUbGBaaGcdaaeWbqaamaaqahabaGaem4uam1aaSbaaSqaaiabdkhaYbqabaGccqGGOaakcuWFYoGygaqbaiabcIcaOiabdMfaznaaBaaaleaacqWGQbGAaeqaaOGaeyOeI0IaemiwaG1aaSbaaSqaaiabdMgaPbqabaGccqGGPaqkcqGGPaqkaSqaaiabdQgaQjabg2da9iabigdaXaqaaiabd6gaUbqdcqGHris5aaWcbaGaemyAaKMaeyypa0JaeGymaedabaGaemyBa0ganiabggHiLdaakiaawUhacaGL9baacqGGSaalaaa@5D79@

where *Y*, *X *and index *i*, *j *are defined in (5).

Since the exponential part in sigmoid function may result in unbounded situation when larger *r *is selected or the data itself may be large, the exponential part was chosen to be controlled by normalizing the data in the following way: denote *Z*_*ji *_= *Y*_*j *_- *X*_*i*_, *i *= 1, ..., *m*, *j *= 1, ..., *n*. *Z *is a pairwise difference matrix between is the disease and normal patients. We then normalize *Z *as *Z*/||*Z*||_2_, where ‖Z‖22=∑i=1pZi2
 MathType@MTEF@5@5@+=feaafiart1ev1aaatCvAUfKttLearuWrP9MDH5MBPbIqV92AaeXatLxBI9gBaebbnrfifHhDYfgasaacPC6xNi=xH8viVGI8Gi=hEeeu0xXdbba9frFj0xb9qqpG0dXdb9aspeI8k8fiI+fsY=rqGqVepae9pg0db9vqaiVgFr0xfr=xfr=xc9adbaqaaeGacaGaaiaabeqaaeqabiWaaaGcbaWaauWaaeaacqWGAbGwaiaawMa7caGLkWoadaqhaaWcbaGaeGOmaidabaGaeGOmaidaaOGaeyypa0ZaaabmaeaacqWGAbGwdaqhaaWcbaGaemyAaKgabaGaeGOmaidaaaqaaiabdMgaPjabg2da9iabigdaXaqaaiabdchaWbqdcqGHris5aaaa@3DC0@ and *p *is the dimension of *Z*.

### Threshold Gradient Direct Regularization (TGDR)

The TGDR approach constructs a parameter path *β*(*λ*) in parameter space that some of the points on that path are close to the point β_* _in(6) representing the optimal solution. The best parameter path will be selected by *k*-fold cross-validation technique. Consider now to minimize

G(β;λ)=argminβ{1−1mn∑i=1m∑j=1nSr(β′(Yj−Xi))+λP(β)},
 MathType@MTEF@5@5@+=feaafiart1ev1aaatCvAUfKttLearuWrP9MDH5MBPbIqV92AaeXatLxBI9gBaebbnrfifHhDYfgasaacPC6xNi=xI8qiVKYPFjYdHaVhbbf9v8qqaqFr0xc9vqFj0dXdbba91qpepeI8k8fiI+fsY=rqGqVepae9pg0db9vqaiVgFr0xfr=xfr=xc9adbaqaaeGacaGaaiaabeqaaeqabiWaaaGcbaGaem4raCKaeiikaGccciGae8NSdiMaei4oaSJae83UdWMaeiykaKIaeyypa0dcbiGae4xyaeMae4NCaiNae43zaCMae4xBa0Mae4xAaKMae4NBa42aaSbaaSqaaiab=j7aIbqabaGcdaGadeqaaKqbakabigdaXiabgkHiTmaalaaabaGaeGymaedabaGaemyBa0MaemOBa4gaaOWaaabCaeaadaaeWbqaaiabdofatnaaBaaaleaacqWGYbGCaeqaaOGaeiikaGIaf8NSdiMbauaacqGGOaakcqWGzbqwdaWgaaWcbaGaemOAaOgabeaakiabgkHiTiabdIfaynaaBaaaleaacqWGPbqAaeqaaOGaeiykaKIaeiykaKIaey4kaSIae83UdWMaemiuaaLaeiikaGIae8NSdiMaeiykaKcaleaacqWGQbGAcqGH9aqpcqaIXaqmaeaacqWGUbGBa0GaeyyeIuoaaSqaaiabdMgaPjabg2da9iabigdaXaqaaiabd2gaTbqdcqGHris5aaGccaGL7bGaayzFaaGaeiilaWcaaa@6AEA@

where *P*(*β*) is the penalties. There are several candidate penalty terms, P1(β)=∑i=1p|βi|
 MathType@MTEF@5@5@+=feaafiart1ev1aaatCvAUfKttLearuWrP9MDH5MBPbIqV92AaeXatLxBI9gBaebbnrfifHhDYfgasaacPC6xNi=xH8viVGI8Gi=hEeeu0xXdbba9frFj0xb9qqpG0dXdb9aspeI8k8fiI+fsY=rqGqVepae9pg0db9vqaiVgFr0xfr=xfr=xc9adbaqaaeGacaGaaiaabeqaaeqabiWaaaGcbaGaemiuaa1aaSbaaSqaaiabigdaXaqabaGccqGGOaakiiGacqWFYoGycqGGPaqkcqGH9aqpdaaeWaqaamaaemaabaGae8NSdi2aaSbaaSqaaiabdMgaPbqabaaakiaawEa7caGLiWoaaSqaaiabdMgaPjabg2da9iabigdaXaqaaiabdchaWbqdcqGHris5aaaa@3F8D@, P2(β)=∑i=1pβi2
 MathType@MTEF@5@5@+=feaafiart1ev1aaatCvAUfKttLearuWrP9MDH5MBPbIqV92AaeXatLxBI9gBaebbnrfifHhDYfgasaacPC6xNi=xH8viVGI8Gi=hEeeu0xXdbba9frFj0xb9qqpG0dXdb9aspeI8k8fiI+fsY=rqGqVepae9pg0db9vqaiVgFr0xfr=xfr=xc9adbaqaaeGacaGaaiaabeqaaeqabiWaaaGcbaGaemiuaa1aaSbaaSqaaiabikdaYaqabaGccqGGOaakiiGacqWFYoGycqGGPaqkcqGH9aqpdaaeWaqaaiab=j7aInaaDaaaleaacqWGPbqAaeaacqaIYaGmaaaabaGaemyAaKMaeyypa0JaeGymaedabaGaemiCaahaniabggHiLdaaaa@3D4B@ and *P*_∞_(*β*) = max_*i *= 1, ..., *p *_*β*_*i*_, where *p *is the number of parameters. We choose the quadratic penalty term P2(β)=∑i=1pβi2
 MathType@MTEF@5@5@+=feaafiart1ev1aaatCvAUfKttLearuWrP9MDH5MBPbIqV92AaeXatLxBI9gBaebbnrfifHhDYfgasaacPC6xNi=xH8viVGI8Gi=hEeeu0xXdbba9frFj0xb9qqpG0dXdb9aspeI8k8fiI+fsY=rqGqVepae9pg0db9vqaiVgFr0xfr=xfr=xc9adbaqaaeGacaGaaiaabeqaaeqabiWaaaGcbaGaemiuaa1aaSbaaSqaaiabikdaYaqabaGccqGGOaakiiGacqWFYoGycqGGPaqkcqGH9aqpdaaeWaqaaiab=j7aInaaDaaaleaacqWGPbqAaeaacqaIYaGmaaaabaGaemyAaKMaeyypa0JaeGymaedabaGaemiCaahaniabggHiLdaaaa@3D4B@ because it is the most common one and use this in our following analysis.

Let *ν *denotes the starting value on the path and Δ*ν *as the increment on the path. To implement the algorithm, we select Δ*ν *= 0.01. For any given *r *of the sigmoid function, a threshold *τ *is varying between 0 and 1. *τ *was chosen in the algorithm to be a vector {0,0.1,0.2,...,0.9,1}. The TGDR algorithm performs the following iteration steps:

• Initialize *β*_*i *_= 0.01 and *ν *= 0, for *i *= 1, ..., p.

• Compute the negative gradient *g*_*i*_(*ν*) = -∂*G*(*β*; *λ*)/∂*β*_*i *_evaluatedat *β*_*i*_(*ν*). Denote *g*_*i*_(*ν*) as the *i*-th component of *g*(*ν*). If *max*_*i*_{|*g*_*i*_(*ν*)|} = 0, stop the iteration.

• Compute vector of *f*_*i*_(*ν*) as the *i*-th component of *f*(*ν*); *f*_*i*_(*ν*) = *I*{|*g*_*i*_(*ν*)| ≥ *τ*·*max*_*l*_|*g*_*l*_(*ν*)|, *l *= 1, ..., *p*}.

• Update *β*_*i*_(*ν *+ Δ*ν*) = *β*_*i*_(*ν*) + Δ*ν *× *g*_*i*_(*ν*) × *f*_*i*_(*ν*). Replace by *ν *+ Δ*ν*.

• Repeat step 2–4 until *β *converges, which means ∑i=1p(βi(k+1)−βi(k))2≤ε
 MathType@MTEF@5@5@+=feaafiart1ev1aaatCvAUfKttLearuWrP9MDH5MBPbIqV92AaeXatLxBI9gBaebbnrfifHhDYfgasaacPC6xNi=xH8viVGI8Gi=hEeeu0xXdbba9frFj0xb9qqpG0dXdb9aspeI8k8fiI+fsY=rqGqVepae9pg0db9vqaiVgFr0xfr=xfr=xc9adbaqaaeGacaGaaiaabeqaaeqabiWaaaGcbaWaaabmaeaacqGGOaakiiGacqWFYoGydaqhaaWcbaGaemyAaKgabaGaeiikaGIaem4AaSMaey4kaSIaeGymaeJaeiykaKcaaOGaeyOeI0Iae8NSdi2aa0baaSqaaiabdMgaPbqaaiabcIcaOiabdUgaRjabcMcaPaaakiabcMcaPmaaCaaaleqabaGaeGOmaidaaaqaaiabdMgaPjabg2da9iabigdaXaqaaiabdchaWbqdcqGHris5aOGaeyizImQae8xTdugaaa@47FF@. *ε *is a pre-select small number and *k *is the number of iteration steps. We choose *ε *= 1 × 10^-8^.

The tuning parameter or the threshold *τ *controls the distribution of estimator β^
 MathType@MTEF@5@5@+=feaafiart1ev1aaatCvAUfKttLearuWrP9MDH5MBPbIqV92AaeXatLxBI9gBaebbnrfifHhDYfgasaacPC6xNi=xH8viVGI8Gi=hEeeu0xXdbba9frFj0xb9qqpG0dXdb9aspeI8k8fiI+fsY=rqGqVepae9pg0db9vqaiVgFr0xfr=xfr=xc9adbaqaaeGacaGaaiaabeqaaeqabiWaaaGcbaacciGaf8NSdiMbaKaaaaa@2D8B@. When *τ *= 0, the estimator *β *is updated on every gradient and therefore the converged estimator is close to ridge regression (RR); while *τ *= 1, the estimated *β *is only updated on the maximum gradients, and the result is roughly corresponding to LASSO (Least Absolute Shrinkage and Selection Operator, [28]). The *τ *values in between 0 and 1 create the estimators more diverse than RR, but less than LASSO.

### Double Cross-Validation

The selection of the parameter path *λ *is determined by *k*-fold cross-validation(CV). Since the empirical AUC is in fact a nonparametric two-sample comparison test, a slight variant of *k*-fold CV is considered and it is called double *k*-fold CV for two samples. The *n *number of diseased patients *Y *is randomly split into roughly equal-sized *K*_1 _parts and *m *number of normal patients *X *into roughly equal-sized *K*_2 _parts. Let *k*_1 _index which of {1, ..., *n*} is in {1, ..., *K*_1_} groups, and *k*_2 _index which of {1, ..., *m*} is in {1, ..., *K*_2_} groups. The cross-validation estimate of prediction error is defined to be

CV(λ)=1−1mn∑i=1m∑j=1nSr(β^′−k1(j)k2(i)(Yj−Xi)),
 MathType@MTEF@5@5@+=feaafiart1ev1aaatCvAUfKttLearuWrP9MDH5MBPbIqV92AaeXatLxBI9gBaebbnrfifHhDYfgasaacPC6xNi=xI8qiVKYPFjYdHaVhbbf9v8qqaqFr0xc9vqFj0dXdbba91qpepeI8k8fiI+fsY=rqGqVepae9pg0db9vqaiVgFr0xfr=xfr=xc9adbaqaaeGacaGaaiaabeqaaeqabiWaaaGcbaqcfaOaem4qamKaemOvayLaeiikaGccciGae83UdWMaeiykaKIaeyypa0JaeGymaeJaeyOeI0YaaSaaaeaacqaIXaqmaeaacqWGTbqBcqWGUbGBaaGcdaaeWbqaamaaqahabaGaem4uam1aaSbaaSqaaiabdkhaYbqabaGccqGGOaakcuWFYoGygaqcgaqbamaaBaaaleaacqGHsislcqWGRbWAdaWgaaadbaGaeGymaedabeaaliabcIcaOiabdQgaQjabcMcaPiabdUgaRnaaBaaameaacqaIYaGmaeqaaSGaeiikaGIaemyAaKMaeiykaKcabeaakiabcIcaOiabdMfaznaaBaaaleaacqWGQbGAaeqaaOGaeyOeI0IaemiwaG1aaSbaaSqaaiabdMgaPbqabaGccqGGPaqkcqGGPaqkaSqaaiabdQgaQjabg2da9iabigdaXaqaaiabd6gaUbqdcqGHris5aaWcbaGaemyAaKMaeyypa0JaeGymaedabaGaemyBa0ganiabggHiLdGccqGGSaalaaa@62B0@

where β^′−k1(j)k2(i)
 MathType@MTEF@5@5@+=feaafiart1ev1aaatCvAUfKttLearuWrP9MDH5MBPbIqV92AaeXatLxBI9gBaebbnrfifHhDYfgasaacPC6xNi=xH8viVGI8Gi=hEeeu0xXdbba9frFj0xb9qqpG0dXdb9aspeI8k8fiI+fsY=rqGqVepae9pg0db9vqaiVgFr0xfr=xfr=xc9adbaqaaeGacaGaaiaabeqaaeqabiWaaaGcbaacciGaf8NSdiMbaKGbauaadaWgaaWcbaGaeyOeI0Iaem4AaS2aaSbaaWqaaiabigdaXaqabaWccqGGOaakcqWGQbGAcqGGPaqkcqWGRbWAdaWgaaadbaGaeGOmaidabeaaliabcIcaOiabdMgaPjabcMcaPaqabaaaaa@39DB@ is calculated by (7) when the *k*_1_(*j*) part of *Y *and *k*_2_(*i*) part of *X *data are removed. The optimal λ* is found by minimizing (8).

The function in (8) reduces the p-dimensional optimization problem to be one-dimensional of minimizing *λ*. The golden section search method [29] was implemented, which does not require the calculation of the derivative, as the optimization method to search for the minimal value between 0 to a large number.

For any given *τ *in {0,0.1,0.2,...,0.9,1}, we run a double *k*-fold cross-validation. Denote *τ*_*l*_, *l *= 1, ..., 11 as the *l*-th component in the vector {0,0.1,0.2,...,0.9,1}. After selection of regularized λ^l
 MathType@MTEF@5@5@+=feaafiart1ev1aaatCvAUfKttLearuWrP9MDH5MBPbIqV92AaeXatLxBI9gBaebbnrfifHhDYfgasaacPC6xNi=xH8viVGI8Gi=hEeeu0xXdbba9frFj0xb9qqpG0dXdb9aspeI8k8fiI+fsY=rqGqVepae9pg0db9vqaiVgFr0xfr=xfr=xc9adbaqaaeGacaGaaiaabeqaaeqabiWaaaGcbaacciGaf83UdWMbaKaadaWgaaWcbaGaemiBaWgabeaaaaa@2F2B@ estimator for given *τ*_*l*_, *l *= 1, ..., 11, the optimal τ^
 MathType@MTEF@5@5@+=feaafiart1ev1aaatCvAUfKttLearuWrP9MDH5MBPbIqV92AaeXatLxBI9gBaebbnrfifHhDYfgasaacPC6xNi=xH8viVGI8Gi=hEeeu0xXdbba9frFj0xb9qqpG0dXdb9aspeI8k8fiI+fsY=rqGqVepae9pg0db9vqaiVgFr0xfr=xfr=xc9adbaqaaeGacaGaaiaabeqaaeqabiWaaaGcbaacciGaf8hXdqNbaKaaaaa@2DAF@ is chosen to be *min*{*CV*(λ^l
 MathType@MTEF@5@5@+=feaafiart1ev1aaatCvAUfKttLearuWrP9MDH5MBPbIqV92AaeXatLxBI9gBaebbnrfifHhDYfgasaacPC6xNi=xH8viVGI8Gi=hEeeu0xXdbba9frFj0xb9qqpG0dXdb9aspeI8k8fiI+fsY=rqGqVepae9pg0db9vqaiVgFr0xfr=xfr=xc9adbaqaaeGacaGaaiaabeqaaeqabiWaaaGcbaacciGaf83UdWMbaKaadaWgaaWcbaGaemiBaWgabeaaaaa@2F2B@), *l *= 1, ..., 11}, where *CV*(*λ*) is evaluated by substituting the regularized λ^l
 MathType@MTEF@5@5@+=feaafiart1ev1aaatCvAUfKttLearuWrP9MDH5MBPbIqV92AaeXatLxBI9gBaebbnrfifHhDYfgasaacPC6xNi=xH8viVGI8Gi=hEeeu0xXdbba9frFj0xb9qqpG0dXdb9aspeI8k8fiI+fsY=rqGqVepae9pg0db9vqaiVgFr0xfr=xfr=xc9adbaqaaeGacaGaaiaabeqaaeqabiWaaaGcbaacciGaf83UdWMbaKaadaWgaaWcbaGaemiBaWgabeaaaaa@2F2B@ in (8). One may also adapt Stone's two-stage cross-validation [30], but it will be computationally intensive.

### Positive constraints

Because of the positive nature of the mass spectrometry data, a positive constraint on the parameter *β *is reasonable. The objective function (7) is minimized subjected to the constraints *β*_*k *_≥ 0 for *k *= 1, ..., *p*. Hence, the estimation β^
 MathType@MTEF@5@5@+=feaafiart1ev1aaatCvAUfKttLearuWrP9MDH5MBPbIqV92AaeXatLxBI9gBaebbnrfifHhDYfgasaacPC6xNi=xH8viVGI8Gi=hEeeu0xXdbba9frFj0xb9qqpG0dXdb9aspeI8k8fiI+fsY=rqGqVepae9pg0db9vqaiVgFr0xfr=xfr=xc9adbaqaaeGacaGaaiaabeqaaeqabiWaaaGcbaacciGaf8NSdiMbaKaaaaa@2D8B@ will result in some exact zero coefficients when the optimization hits the positive constraint boundary, which means that the biomarker has no contribution to maximize the AUC.

## Authors' contributions

JY implemented the software for the mass spectrometry preprocessing method and threshold gradient direct regularization and area under the curve and drafted the manuscript. HL designed the TGDR-AUC algorithm and helped draft the manuscript. CK performed the mass spectrometry glycomics experiment and helped revise the manuscript. CBL participated in the design of the mass spectrometry glycomics experiment. DMR contributed to the methods of preprocessing the mass spectrometry spectrum and oversaw the overall project. All authors read and approved the final manuscript.

## Supplementary Material

Additional file 1 Simulated data with different combinations of *n *and *p *used to evaluate TGDR-AUC algorithm. Click here for file

Additional file 2 Introduction on how to use the provided C++ code and the simulated data. Click here for file

Additional file 3 C++ source code for TGDR-AUC algorithm to estimate optimal parameters of *λ *and *τ*. Click here for file

Additional file 4 C++ source code for TGDR-AUC algorithm to estimate linear combination parameters and AUC after selecting optimal *λ *and *τ*. Click here for file
