# Supplementary material for: On the analysis of glycomics mass spectrometry data via the regularized area under the ROC curve
Source: BMC Bioinformatics. 2007 Dec 12;8:477. doi: 10.1186/1471-2105-8-477 (PMC2211327; doi:10.1186/1471-2105-8-477)
Supplement: Additional file 2 — Introduction on how to use the provided C++ code and the simulated data. [file 1471-2105-8-477-S2.doc]

READ ME

1. The simulated data contains 12 files. The file names indicate number of biomarker and number of sample size combinations. For example, simup10n50.txt means the generated data contains 10 numbers of biomarkers and 50 normal samples and 50 cancer samples. In each data file, each row of the data means one sample. The first column indicates the status of the sample, -1 for normal and 1 for cancer. The rest columns are generated intensities for mass spectrometry data and each column represents one m/z value.
2. simu_golden.cpp is the C++ code for TGDR-AUC algorithm with 3-fold cross-validation and golden section search to selection the optimal parameters$\lambda$and$\tau$. The algorithm is written in the case of “simup10n50.txt”. If you want to run other files, you need to change three things: a. nn, the sample size number; b. pp, the number of biomarker; c. name of the file. These three changes are highlighted where “/*** */” are in the code. The algorithm will generate an output .txt for user to process optimal parameters. Note: the data file and simu_golden.cpp need to under the same folder.
3. simu_final_model.cpp is the C++ code for TGDR-AUC algorithm after training the optimal parameters$\lambda$and$\tau$. The code is again for the case of “simup10n50.txt”. If the user wants to use for another file, please change the three things listed in 2. Additionally, please also change in the algorithm the optimal$\lambda$and$\tau$selections, which are also highlighted with“/*** */”. The output file will give the AUC estimation. Note: the data file and simu_final_model.cpp need to under the same folder.
